# Patient-reported outcome measures for paediatric gender-affirming care: A systematic review

**DOI:** 10.1093/pch/pxae019

**Published:** 2024-07-24

**Authors:** Liam Jackman, Cynthia Chan, Chloë Jacklin, Eve Deck, Ann C Lee, Melissa Stepney, Conrad Harrison, Abhilash Jain, Jeremy Rodrigues, Rakhshan Kamran

**Affiliations:** Temerty Faculty of Medicine, University of Toronto, Toronto, Ontario; Department of Family Medicine, University of Ottawa, Ottawa, Ontario; Medical Sciences Division, University of Oxford, Oxford, UK; Department of Family Medicine, Western University, London, Ontario; Faculty of Medicine, University of Ottawa, Ottawa, Ontario; Department of Psychiatry, University of Oxford, Oxford, UK; Nuffield Department of Orthopaedics, Rheumatology and Musculoskeletal Sciences, University of Oxford, Oxford, UK; Nuffield Department of Orthopaedics, Rheumatology and Musculoskeletal Sciences, University of Oxford, Oxford, UK; Warwick Clinical Trials Unit, University of Warwick and Department of Plastic Surgery, Stoke Mandeville Hospital, Buckinghamshire Healthcare NHS Trust, Aylesbury, UK; Nuffield Department of Orthopaedics, Rheumatology and Musculoskeletal Sciences, University of Oxford, Oxford, UK

**Keywords:** *Gender identity*, *Health services for transgender persons*, *Patient-reported outcome measures*, *Transgender persons*

## Abstract

**Objectives:**

Patient needs must be comprehensively measured to offer paediatric gender-affirming care in line with clinical standards. Patient-reported outcome measures (PROMs) are self-report tools that measure outcomes deemed to be of importance to patients. PROMs may assess a single outcome or multiple outcomes simultaneously, such as symptoms, functional ability, and quality of life. This study aims to identify PROMs for paediatric gender-affirming care.

**Methods:**

This systematic review is PRISMA-compliant and was prospectively registered on PROSPERO (CRD42023461959). Six databases were searched: PubMed, Embase, MEDLINE, PsycINFO, CINAHL, and Web of Science from inception to December 16, 2022. Articles meeting the following criteria were included: 1) Original article; 2) Administers a formally-developed PROM; 3) Focuses on gender-affirming care; and 4) Focuses on paediatric populations. Screening and data extraction occurred independently and in duplicate. Data extracted include study/demographic information, and details of PROM used.

**Results:**

In total, 20 articles were included, representing a total of 5793 paediatric patients undergoing gender-affirming care. Most studies (13, 65%) focused on hormonal gender-affirming care. A total of 38 different PROMs for paediatric gender-affirming care were identified, ranging from 4 to 120 items each (mean 23 items; median 14 items). Most PROMs (n = 22) measured psychological functioning, with eight PROMs measuring quality of life, and three PROMs measuring gender-related concepts (i.e., gender dysphoria/euphoria). Commonly used PROMs include the Utrecht Gender Dysphoria Scale (n = 4; 20%), Body Image Scale (n = 5;25%), and Youth Self-Report (n = 8; 40%).

**Conclusions:**

A total of 38 PROMs were identified measuring a range of concepts for paediatric gender-affirming care.

Gender-affirming care is a term used to refer to a broad range of psychosocial, hormonal, and surgical care to assist with a person’s gender transition (1). In recent years, transgender and gender diverse (TGD) youth have become more visible, yet increasingly are subject to societal hostilities (2). There has been a possible stagnation of affirmative healthcare internationally. TGD youth are facing daily challenges of exhaustion and fatigue due to living within societies that do not fully support their gender identity. There have also been increases in the number of patients seeking gender-affirming care (2,3). Globally, clinical guidelines and international standards outline that individual patient perspectives must be comprehensively understood to offer effective gender-affirming care (4). Despite the presence of clinical guidelines, several reports outline limitations with clinician knowledge and competence for providing effective gender-affirming care to TGD youth, which adversely impacts care (5–10). Patient-reported outcome measures (PROMs) offer a potential solution to improve the quality of paediatric gender-affirming care by allowing for the measurement of individual patient perspectives on the concepts that matter most to them (11,12).

PROMs are standardized self-report instruments that measure health concepts that matter most to patients from their own perspective (13). PROM use in clinical practice can allow for the detection of clinical issues which may otherwise go unaddressed, enhance patient–provider communication, increase patient satisfaction, and improve care outcomes (14–16), but many clinicians are unaware of these benefits (17). Not knowing which PROMs to use, nor how to effectively administer them in clinical encounters limits the success of their implementation (18–20). Past literature emphasizes the potential for PROM implementation to improve gender-affirming care quality if used effectively (21).

The aim of this systematic review was to identify PROMs used in the literature for paediatric gender-affirming care.

## METHODS

This systematic review was prospectively registered on PROSPERO (CRD42023461959) in September 2023 and follows Preferred Reporting Items for Systematic Reviews and Meta-Analysis (PRISMA) guidance (22). This study was reviewed by the Clinical Trials and Research Governance Body at the University of Oxford who deemed this study exempt from ethics approval as there was no human participant data collection.

### Patient and public involvement

Seven TGD individuals (including young adults) were involved in this systematic review including confirming the relevance and importance of this systematic review, aiding with search strategy development, and confirming the relevance, applicability, and importance of findings. Upon completion of the systematic review, results were presented to patients and public partners, and their recommendations were used to inform the discussion. For example, patients and public partners highlighted that it was important to discuss confidentiality and data security with respect to PROM responses. Patients and public partners were recruited from national transgender charity organizations and community support groups.

### Eligibility criteria

Articles meeting the following inclusion criteria were included in this review: 1) Original article; 2) Administers a formally-developed PROM; 3) Focuses on gender-affirming care; and 4) Focuses on paediatric populations. There were no restrictions on language or date of publication.

### Information sources

The following six databases were searched independently from inception to December 15, 2022: PubMed, Embase, MEDLINE, PsycINFO, CINAHL, and Web of Science. Grey literature was searched through grey literature databases (i.e., opengrey.eu), customized Google searching, and targeted searching of relevant TGD healthcare websites (detailed in Supplementary Appendix 1). Seven patient and public partners representing individuals from the TGD community, and an information scientist specializing in systematic review search strategies were involved in creating and reviewing the search strategy. The search strategy was also used in a separate systematic review conducted by our team on PROM implementation for gender-affirming care (12).

### Search strategy

The search strategy is available in Supplementary Appendix 1.

### Selection process

Title and abstract screening, and full-text screening occurred independently and in duplicate on the Covidence platform (LJ, CC, CJ, ED, ACL) with conflicts resolved by a third reviewer (RK).

### Data collection process

Data extraction occurred independently and in duplicate (LJ, CC, CJ, ED, ACL) with conflicts resolved by a third reviewer (RK).

### Data items

Extracted data items include study information (study title, year of publication), number of patients, age, country patients were recruited from, race/ethnicity of included patients, gender-affirming care provided, level of evidence according to the Oxford Centre for Evidence-Based Medicine (23), PROMs used, length of PROMs, timing of PROM administration, location of PROM completion, mode of PROM administration (i.e., online, by post), data collection platform used for PROMs administered online, frequency of PROM administration, data security provisions for PROMs, how PROM results were displayed, and mention of PROM training for staff.

### Study risk of bias assessment

The Critical Appraisal Skills Programme (CASP) Tool (24) was used to assess the quality of studies independently and in duplicate (LJ, CC, CJ, ED, ACL) with conflicts resolved by a third reviewer (RK).

### Synthesis methods and reporting bias assessment

The Synthesis Without Meta-Analysis guideline was used to report data synthesis (25). Content analysis for each PROM identified in this systematic review used guidance from the Patient-Reported Outcomes Measurement Information System (PROMIS) (26). Each PROM was categorized according to the health concept measured (i.e., psychological functioning), using guidance from PROMIS. A consensus meeting to review coding and ensure consistency was held (LJ, CC, ED, ACL, RK).

### Statistical analysis

Descriptive frequencies were calculated for demographic study information and patient characteristics. Content analysis for health concepts measured by PROMs was narratively synthesized and did not use quantitative statistical techniques.

## RESULTS

### Study selection

**Figure 1.**
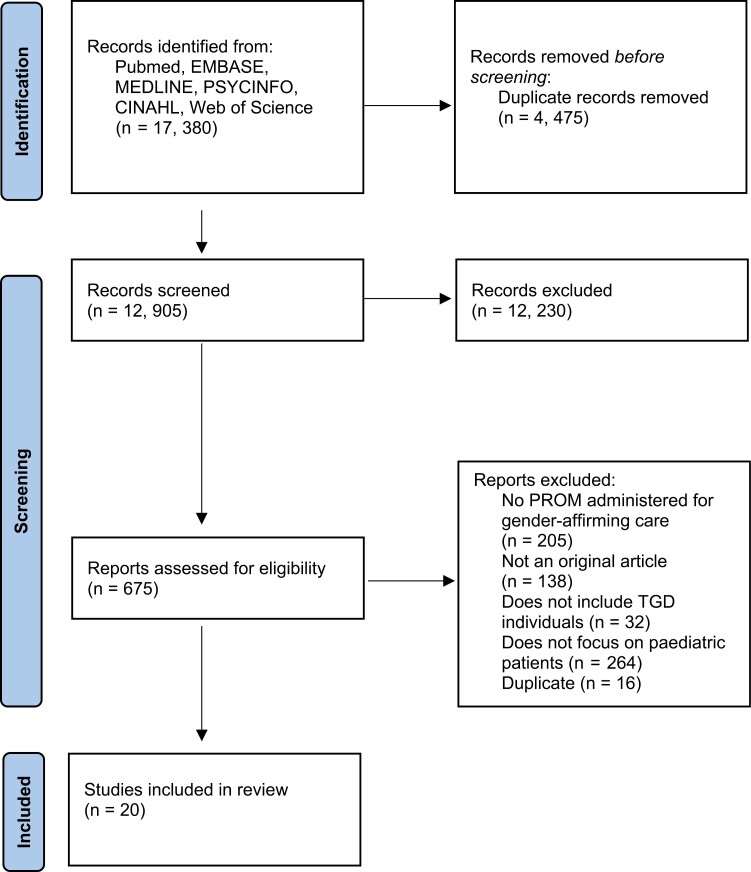
PRISMA diagram of study selection.

A total of 17,380 records were identified from the search strategy, with 4475 duplicate records removed prior to screening. A total of 12,905 records underwent screening with 20 studies included in the systematic review (27–46) (Figure 1).

### Study characteristics

A total of 5793 TGD paediatric patients from nine countries were represented in the 20 studies. The majority of articles were from: the USA (n = 8), the Netherlands (n = 3), and Germany (n = 3). Of the six studies (30%) reporting race and ethnicity, most participants were White (95%). Most studies (n = 13; 65%) focused on hormonal therapy. Most studies (n = 14; 70%) were ranked as 2c (outcomes research), with remaining studies (n = 6; 30%) ranked as 2b (cohort studies) according to the Oxford Centre for Evidence-Based Medicine Level of Evidence guidance (23). Table 1 provides a complete overview of the study and patient characteristics for included studies in this systematic review. Supplementary Appendix 2 outlines the age distribution of paediatric patients sorted by included studies.

**Table 1.** Demographic information of included articles

	n (%) studies
Country	
USA	8 (40)
Germany	3 (15)
The Netherlands	3 (15)
Australia	1 (5)
Belgium, The Netherlands, UK	1 (5)
Brazil	1 (5)
Canada, the Netherlands	1 (5)
Switzerland	1 (5)
UK	1 (5)
	n (%)
Race (n = 6 studies reporting race and ethnicity)	
White	544 (95.4)
African American	10 (1.8)
Multiracial	12 (2.1)
American Indian or Alaska Native	1 (0.2)
Asian or Pacific Islander	3 (0.5)
Ethnicity (n = 6 studies reporting race and ethnicity)	
Hispanic	71 (15.2)
Non-Hispanic	397 (84.8)
	n (%) studies
Gender-affirming care provided	
Hormonal	13 (65)
Hormonal and psychosocial	3 (15)
Hormonal and surgical	1 (5)
Hormonal, psychosocial, surgical	1 (5)
Voice therapy	2 (10)

### PROMs for paediatric gender-affirming care

In the 20 included studies, 38 different PROMs were administered, ranging from 4 to 120 items each (mean 23 items; median 14 items). The number of PROMs administered per study ranged from 1 to 8 PROMs (mean 3 PROMs; median 2 PROMs). The total number of items administered to paediatric patients per study ranged from 12 to 198 items (mean 89 items; median 95 items). Most PROMs (n = 22) measured psychological functioning, with eight PROMs measuring global quality of life, and three PROMs measuring gender-related concepts (i.e., gender dysphoria/euphoria). The most commonly used PROMs were: the Utrecht Gender Dysphoria Scale (n = 4; 20%) (47), the Body Image Scale (n = 5; 25%) (48), and the Youth Self-Report (n = 8; 40%) (49). Table 2 provides an overview of the PROMs identified from this study, the number of items per PROM, target population, mode of administration, original language, translations available, licencing information, and their frequency of use, organized by construct measured.

**Table 2.** Overview of PROMs for paediatric gender-affirming care

PROM used in paediatric gender-affirming care	Number of items	Target population	Mode of administration (e.g., self-report, interview-based, patient/proxy report, etc.)	Original language	Translations	Licencing information	Number of studies using PROMs (n, %)
PROMs measuring gender-related concepts (i.e., gender dysphoria/euphoria)							
Gender Distress Scale (50)	14	Youth age puberty—17 attending clinics for puberty blockers or gender-affirming hormone therapy	Interview-based	English	French	https://transyouthcan.ca/wp-content/uploads/2021/04/Gender-Distress-Scale-vSHARE_EN-2021.pdf	1 (5) (46)
Gender Positivity Scale (51)	11	Youth age puberty—17 attending clinics for puberty blockers or gender-affirming hormone therapy	Interview-based	English	French	https://transyouthcan.ca/wp-content/uploads/2021/04/Gender-Positivity-Scale-vSHARE_EN-2021.pdf	1 (5) (46)
Utrecht Gender Dysphoria Scale (52)	12	Adolescents with gender dysphoria	Self-report	English	NR	NR	4 (20) (33–35,39)
PROMs measuring quality of life							
Child Health Questionnaire-Child Form (CHQ-CF87) (53)	87	Children 10 and older, not condition-specific	Self-report	English	25 languages, available at: http://www.healthact.com/translation-chq.php	http://www.healthact.com/translation-chq.php	1 (5) (43)
Kidscreen-27 (54)	27	Ages 8–18, not condition-specific	Self-report	English	Over 13 languages, available at: https://www.kidscreen.org/english/questionnaires/language-versions/	https://www.kidscreen.org/english/questionnaires/language-versions/	1 (5) (31)
Kidscreen Subscales Autonomy and Parental Relationship (55)	11	Ages 8–18, not condition-specific	Self-report	English	Over 13 languages, available at: https://www.kidscreen.org/english/questionnaires/language-versions/	https://www.kidscreen.org/english/questionnaires/kidscreen-52/	1 (5) (32)
PedsQL 4.0 Generic Core Scale 4.0 Child Self Report (56)	23	Ages 8–12, not condition-specific	Self-report	English	Multiple languages, available at: https://www.pedsql.org/translations.html	https://www.pedsql.org/conditions.html	1 (5) (44)
PedsQL General Well-Being Scale for ages 8–25 (56)	6	Ages 8–25, not condition-specific	Self-report	English	Multiple languages, available at: https://www.pedsql.org/translations.html	https://eprovide.mapi-trust.org/instruments/pedsql-general-well-being-scale	1 (5) (44)
Quality of Life Enjoyment and Satisfaction Questionnaire (QLES-Q-SF) (57)	15	Ages 6–17, not condition-specific	Self-report	English	NR	https://www.phenxtoolkit.org/protocols/view/180303#:~:text=The%20Pediatric%20Quality%20of%20Life,to%205%20(Very%20Good).	1 (5) (37)
Satisfaction with Life Scale (58)	5	Children 10 and older, not condition-specific	Self-report	English	German	http://labs.psychology.illinois.edu/~ediener/ScalesforChildren.html	1 (5) (34)
WHOQOL-BREF (59)	24	Adults 18 and older, not condition-specific, used but not yet validated for children	Self-report	English	Multiple languages, available at: https://www.who.int/tools/whoqol/whoqol-bref	http://depts.washington.edu/seaqol/WHOQOL-BREF	1 (5) (34)
PROMs measuring psychological and physical functioning							
SF-8 (60)	8	Adolescents and adults 14 and older, not condition-specific	Self-report	English	Over 30 languages	NA	1 (5) (31)
PROMs measuring psychological functioning							
13-item Level 2 PROMIS Emotional Distress-Anxiety Scale for ages 11-17 (61)	13	Ages 11–17, anxiety	Self-report	English	Multiple languages, available at: https://www.healthmeasures.net/explore-measurement-systems/promis/intro-to-promis/available-translations	https://www.healthmeasures.net/explore-measurement-systems/promis/obtain-administer-measures	1 (5) (27)
14-item Level 2 PROMIS Emotional Distress-Depression Scale for ages 11-17 (61)	14	Ages 11–17, depression	Self-report	English	Multiple languages, available at: https://www.healthmeasures.net/explore-measurement-systems/promis/intro-to-promis/available-translations	https://www.healthmeasures.net/administrator/components/com_instruments/uploads/PROMIS%20Depression%20Scoring%20Manual_08Sept2023.pdf	1 (5) (27)
Beck Depression Inventory (62)	21	Ages 13 and older, depression	Self-report	English	Spanish, Chinese, Dutch, Finnish, French (Canadian), German, Korean, Polish, Swedish, Arabic, Turkish	https://www.pearsonassessments.com/store/usassessments/en/Store/Professional-Assessments/Personality-%26-Biopsychosocial/Beck-Depression-Inventory/p/100000159.html	2 (10) (34,39)
Body Image Scale (48)	30	Ages 17 and older, transgender populations, used but not yet validated for children	Self-report	English	NR	NA	5 (25) (32,38,39,42,45)
Children’s Depression Inventory (63)	28	Ages 7–17, depression	Self-report	English	Spanish	https://www.pearsonassessments.com/store/usassessments/en/Store/Professional-Assessments/Personality-%26-Biopsychosocial/Children%27s-Depression-Inventory-2/p/100000636.html	1 (5) (38)
Eating Disorder Examination Questionnaire (EDE-Q) (64)	28	Ages 14 and older, eating disorders	Self-report	English	Multiple languages, available at: https://www.corc.uk.net/outcome-experience-measures/eating-disorder-examination-questionnaire-ede-q/	https://www.corc.uk.net/outcome-experience-measures/eating-disorder-examination-questionnaire-ede-q/	1 (5) (36)
German Version of 1991 Youth Self-Report (65)	120	Children and adolescents with a psychiatric diagnosis	Self-report	German	NR	NR	1 (5) (31)
Lowenberg and Krege (66) (Unnamed) 2008	NR	TGD individuals with gender-affirming surgery treated with psychotherapy	Self-report	German	NR	NR	1 (5) (29)
Liebowitz Social Anxiety Scale (LSAS) (67)	24	Ages 13 and older, social anxiety	Self-report	English	NR	https://nationalsocialanxietycenter.com/liebowitz-sa-scale/	1 (5) (38)
Modified Depression Scale (MDS) (68)	6	Ages 13 and older, depressive symptoms	Self-report	English	NR	NR	1 (5) (46)
Overall Anxiety Severity and Impairment Scale (OASIS) (69)	5	Adults 18 and older, anxiety, used but not yet validated for children	Self-report	English	NR	NR	1 (5) (46)
Patient Health Questionnaire (PHQ-9) Modified for Teens (70)	9	Ages 11–17, depression	Self-report	English	NR	NR	1 (5) (37)
PROMIS Anxiety (71)	8	Ages 8–17, anxiety	Self-report	English	Multiple languages, available at: https://www.healthmeasures.net/explore-measurement-systems/promis/intro-to-promis/available-translations	https://commonfund.nih.gov/promis/index	1 (5) (44)
PROMIS Depression (72)	8	Ages 8–17, depression	Self-report	English	Multiple languages, available at: https://www.healthmeasures.net/explore-measurement-systems/promis/intro-to-promis/available-translations	https://commonfund.nih.gov/promis/index	1 (5) (44)
Quick Inventory of Depressive Symptoms (73)	16	Adults 18 and older, depression, used but not yet validated for children	Self-report	English	NR	https://eprovide.mapi-trust.org/instruments/self-report-quick-inventory-of-depressive-symptomatology	1 (5) (42)
Suicide Behaviours Questionnaire-Revised (74)	4	Ages 13 and older, suicidal behaviour	Self-report	English	NR	NR	1 (5) (38)
Screen for Child Anxiety Related Disorders (75)	41	Ages 8–11, anxiety	Self-report	English	French, German, Italian, Dutch, Spanish, Chinese, Arabic, Thai	https://www.psychiatry.pitt.edu/research/investigator-resources/assessment-instruments	2 (10) (38,42)
Spielberger’s Trait Anger (76)	35	Ages 9–18, anger	Self-report	English	NR	https://paa.com.au/product/staxi-2-ca/#:~:text=The%20STAXI%2D2%20C%2FA,aspects%20of%20anger%20in%20children.	2 (10) (34,39)
Spielberger’s Trait Anxiety (77)	20	Ages 9–12, anxiety	Self-report	English	Multiple languages, available at: https://www.mindgarden.com/contact-us	https://www.mindgarden.com/146-state-trait-anxiety-inventory-for-children#horizontalTab4	2 (10) (34,39)
Subjective Happiness Scale (78)	4	Adults 18 and older, happiness, used but not yet validated for children	Self-report	English	NR	https://ppc.sas.upenn.edu/sites/default/files/subjectivehappinessscale.pdf	1 (5) (34)
The Center for Epidemiologic Studies Depression Scale (CESD-R) (79)	20	Ages 8 and older	Self-report	English	Arabic, Bhutanese, Chinese, Croatian, French, German	https://cesd-r.com/	1 (5) (37)
Youth Self-Report (49)	102	Ages 11–18, behavioural and emotional symptoms	Self-report	English	Spanish, French, Tagalog, Vietnamese, Chinese, American Sign Language, Farsi, Polish, Russian, Urdu	https://www.nctsn.org/measures/youth-self-report-11-18	8 (40) (28,29,32–34,39,41,45)
PROMs measuring satisfaction with treatment							
Client Satisfaction Questionnaire (ZUF-8) (80)	8	Age NR, patient satisfaction	Self-report	German	NR	http://www.gfqg.de/downloads.html?file=files/content/downloads/Assessment/ZUF-8%20Fragebogen.pdf	1 (5) (30)
PROMs measuring severity of insomnia							
Insomnia Severity Index (81)	7	Ages 17 and above, insomnia symptoms	Self-report	English	Multiple languages, available at: https://eprovide.mapi-trust.org/instruments/insomnia-severity-index	https://eprovide.mapi-trust.org/instruments/insomnia-severity-index	1 (5) (44)
PROMs measuring social functioning							
Short Form of Perceived Social Support Questionnaire (F-SozU) (82)	14	Ages 14 and older, social support	Self-report	German	NR	NR	1 (5) (30)
PROMs measuring voice functioning							
Transsexual Voice Questionnaire MtF (83)	30	Adults 18 and older, TGD individuals undergoing voice therapy, used but not yet validated for children	Self-report	English	Multiple languages	https://www.latrobe.edu.au/__data/assets/pdf_file/0010/1393363/TGV-Resources.pdf	1 (5) (40)

Studies administered the same PROMs between 1 and 4 times per study (mean 1.7; median 1.5). Of the 20 studies included in this systematic review, 10 administered PROMs as part of the baseline assessment for gender-affirming care only. A total of seven studies administered PROMs as part of a baseline assessment and one follow-up, a single study administered a PROM as part of a baseline assessment and two follow-ups, and a single study administered PROMs as part of a baseline assessment and three follow-ups. One study divided PROM administration into three phases: pre-treatment, during treatment, and post-treatment.

Paediatric patients most often completed PROMs in the clinic (n = 12; 60%), with some studies reporting patients completing PROMs at home before their appointment (n = 3; 15%). The remaining studies (n = 5; 25%) did not report the location of PROM completion. A total of three studies (15%) described using online PROMs with their paediatric patients, and two studies (10%) mailed PROMs to patient homes. For the three studies using an online platform to administer PROMs, two used the Qualtrics platform and one used the REDCap platform.

No studies reported on data security measures to securely handle PROM data. When displaying PROM results, the most common method was using the mean score (n = 14; 70%), with some studies using a graph to display scores over time (n = 3; 15%), or a table displaying PROM scores (n = 3; 15%).

### Risk of bias

CASP checklists (24) were used for the risk of bias assessment for this study. All studies in this review addressed a clearly focused issue and demonstrated acceptable recruitment. However, no studies considered all relevant confounding factors or took into account confounding factors in their design or analysis. The follow-up of subjects was also limited in half of the studies (Supplementary Appendix 3).

## DISCUSSION

This systematic review identifies PROMs currently used in paediatric gender-affirming care. Compared to our previous systematic review focusing on PROMs and ad hoc instruments used in adult gender-affirming care (12), fewer PROMs were identified in paediatric gender-affirming care settings. The burden of PROM administration was greater in paediatric settings relative to adult settings as generally, patients were asked to complete more items. In our previous systematic review, which considered PROMs and ad hoc instruments, a mean of 57 items were administered for adult gender-affirming care, and a mean of 116 items for paediatric gender-affirming care (12). The present study considering PROMs (and not ad hoc instruments) identified a mean of 89 items for paediatric gender-affirming care. Results from this systematic review can be used practically by clinicians to select a PROM to use for their paediatric gender-affirming care setting, lead outcome measurement and evaluation initiatives, and guide implementation efforts. This study also helps fill a knowledge gap for clinicians who may not have an understanding of the PROMs available for paediatric gender-affirming care and how they may be used to enhance the quality of care.

Table 2 provides relevant information for PROMs currently used in paediatric gender-affirming care, which can be useful to clinicians and researchers planning to implement PROMs in their settings. A practical approach to selecting a PROM should include first identifying which concept one is interested in measuring (e.g., gender-related concepts, psychological functioning, or quality of life). Once the concept is identified, Table 2 can be used to provide an overview of PROMs available to measure the concept of interest. Further considerations to select a PROM within a concept of interest include identifying the PROM which has a target population in line with the patient population of interest (i.e., some PROMs like the Gender Distress Scale (50) are targeted for youth from puberty age to 17, while the CESD-R, which measures depression, is targeted for ages 8 and older). Other important considerations for PROM selection include whether the PROM is offered in a relevant translation (i.e., the Gender Distress Scale (50) is offered in a French translation), and if there is a financial cost for licencing the PROM (this information is linked in Table 2 under the heading ‘licencing information’). Finally, it is important to also select a PROM which is meaningful and relevant for the target patient population. Presenting a few PROM options to a patient and public involvement group at a clinic and collecting feedback on which PROM is most acceptable can help to guide selection. A common piece of feedback from patients is that they prefer a PROM which has minimal patient burden (i.e., low number of items) (84). If a PROM demonstrates a high number of items, one strategy to make the PROM shorter while retaining validity and reliability is applying techniques from computerized adaptive testing (85).

We have not identified any systematic reviews published or registered on PROSPERO on patient-reported outcome measurement for paediatric gender-affirming care, despite several calls for more research on outcome measurement for paediatric gender-affirming care (86–88). The results from the present systematic review fill this gap in the literature and provide practical information which may be helpful to guide PROM selection and use for paediatric gender-affirming care.

The sample of participants included in this review demonstrates a lack of diversity. This is in line with other research in gender-affirming care which also mentions the lack of non-White participants in gender-affirming care research (7,89,90). It is important for future research on paediatric gender-affirming care to include participants representing diverse backgrounds (e.g., neurodiverse, young people of colour) to ensure the perspectives and experiences of racial and ethnic minority patients are represented (91,92). It is also important to ensure that the language used for paediatric PROMs considers vocabulary, comprehension, and the age at which children can provide responses to the PROM that are reliable and valid; which can be investigated through qualitative studies (93,94). An example of a PROM which may require modification to be appropriate for a North American paediatric population is the Lowenberg and Krege instrument (66), which was originally developed in Germany. An instrument which may have widespread utility is the Gender Distress Scale (50), available in both English and French.

Patient confidentiality is important for paediatric gender-affirming care, especially if the patient is not ‘out’ to family members. Despite the importance of patient confidentiality in this clinical area, this systematic review did not identify any studies reporting on measures for the secure use and storage of PROM data. This is a particularly relevant area for future PROM initiatives in paediatric gender-affirming care, as being unable to communicate with patients about how their PROM data will be securely stored/handled may impact their willingness to complete PROMs (95–97). Patient and public partners involved in this systematic review also emphasize that data security of PROMs and communication on how results will be kept confidential is key for patient engagement. It is also important for healthcare professionals providing gender-affirming care to understand that PROM responses may be affected by environmental and interpersonal dynamics. For example, TGD youth may not have parental support, and therefore parental presence during PROM administration and completion may impact the patients’ responses (98).

As this systematic review identified variation in PROMs used and concepts measured, there is a demonstrated need to standardize concepts measured in paediatric gender-affirming care. As the process of developing new PROMs is time and resource-intensive and may contribute to research waste, several recommendations have been made to refine existing PROMs to meet current standards rather than create entirely new PROMs (99–101). The results from this systematic review can be used to help guide consensus efforts in standardizing PROM administration for paediatric gender-affirming care, and guide efforts to refine existing PROMs identified to meet current needs. This will help to minimize future research waste. When implementing a PROM identified from this study, past research emphasizes that PROM implementation for gender-affirming care must consider including patient educational information about the PROM, considering accessibility options (i.e., large font size versions), and communication with patients on how PROMs can be used to improve care (102).

Strengths of this systematic review include working in partnership with patients and public involvement to confirm the relevance and applicability of this research. Further, this systematic review identifies all PROMs currently in use for paediatric gender-affirming care and uses established guidance from PROMIS to categorize the concepts measured. Paediatricians can use the PROMs identified in Table 2 in their clinical setting to better capture patient perspectives, measure treatment outcomes (tracking PROM scores over time to see if there is improvement in the concept of interest, such as gender dysphoria, being measured), and even compare outcomes between different treatments (i.e., use PROM results to see if certain treatments provide patients better outcomes than others). The use of PROMs can help to supplement and augment clinical encounters, providing quantitative data which can help guide decision-making. Discussion of PROM results with patients can also help to improve shared decision-making when deciding on the next steps of treatment, and showing patients how their results have changed over the course of treatment.

Limitations of this systematic review include the risk that some articles might have been missed despite the comprehensive search. Secondly, this systematic review did not perform a COSMIN analysis of the PROMs identified as this was beyond the scope of this review (103). COSMIN analysis includes a rating of the measurement properties (i.e., internal consistency, reliability, content validity, measurement error) of identified PROMs based on development studies for each PROM (this includes performing separate systematic searches for each PROM to identify its development studies) (103). Future researchers can use the results from this systematic review to perform a COSMIN analysis of the PROMs identified. A practical way to overcome this limitation is for a clinician/researcher to find a PROM they are interested in (from Table 2), review the paper/papers involved in developing the PROM, and check to see if the measurement properties of the instrument meet COSMIN criteria (103). Thirdly, due to the heterogeneity in reporting gender, the gender of participants in this systematic review was unable to be synthesized. Future research should seek to use the two-step method (104) to ensure standardization when reporting on gender. Fourthly, there was also heterogeneity in the age ranges for patients in included studies, with some of the upper ranges above the paediatric range. As some studies did not report on the range, SD, or IQR for age ranges, there is a general need for more rigour in age reporting for paediatric gender-affirming care research.

## CONCLUSION

This systematic review identifies PROMs used for paediatric gender-affirming care, organized by the concepts they measure and patient burden, which serves as a practical guide to help clinicians and researchers select a PROM to use for their setting and help guide implementation efforts.

## Supplementary Material

pxae019_suppl_Supplementary_Appendixs
