# Supplementary material for: Patient-reported outcome measures for paediatric gender-affirming care: A systematic review
Source: Paediatr Child Health. 2024 Jul 24;29(8):514–27. doi: 10.1093/pch/pxae019 (PMC11840252; doi:10.1093/pch/pxae019)
Supplement: pxae019_suppl_Supplementary_Appendixs [file pxae019_suppl_supplementary_appendixs.pdf]

Patient-Reported Outcome Measures for Paediatric Gender-Affirming Care: A Systematic Review

Appendix

Appendix 1. Search Strategy

This search was a part of a larger systematic review our team has conducted[12]

The rows in each table below were combined with the AND Function

PUBMED
(((((((("Patient Outcome Assessment"[Mesh] OR HR-PRO[tiab] OR HRPRO[tiab] OR HRQL[tiab] OR HRQoL[tiab] OR QL[tiab] OR QoL[tiab] OR quality of life[tw] OR life quality[tw] OR health index*[tiab] OR health indices[tiab] OR health profile*[tiab] OR health status[tw] OR ((patient[tiab] OR self[tiab] OR child[tiab] OR parent[tiab] OR carer[tiab] OR proxy[tiab]) AND ((report[tiab] OR reported[tiab] OR reporting[tiab]) OR (rated[tiab] OR rating[tiab] OR ratings[tiab]) OR based[tiab] OR (assessed[tiab] OR assessment[tiab] OR assessments[tiab]))) OR ((disability[tiab] OR function[tiab] OR functional[tiab] OR functions[tiab] OR subjective[tiab] OR satisfaction[tiab] OR implementation[tiab] OR health services[tiab] OR utility[tiab] OR utilities[tiab] OR wellbeing[tiab] OR well being[tiab]) AND (index[tiab] OR indices[tiab] OR instrument[tiab] OR instruments[tiab] OR measure[tiab] OR measures[tiab] OR questionnaire[tiab] OR questionnaires[tiab] OR profile[tiab] OR profiles[tiab] OR scale[tiab] OR scales[tiab] OR score[tiab] OR scores[tiab] OR status[tiab] OR survey[tiab] OR surveys[tiab] OR PROM [tiab])))))
("Transsexualism"[Mesh] OR "Transvestism"[Mesh] OR "Transgender Persons"[Mesh] OR "Sex Reassignment Procedures"[Mesh] OR "Health Services for Transgender Persons"[Mesh] OR "Gender Identity"[Mesh] OR “Gender Dysphoria”[Mesh]) OR (transgender*[tw] OR transsexual*[tw] OR "gender identit*"[tw] OR "sex reassignment*"[tw] OR "gender dysphori*"[tw] OR “gender euphori*”[tw] OR "trans men"[tw] OR "trans man"[tw] OR "trans women"[tw] OR "trans woman"[tw] OR "cross gender*"[tw] OR crossgender*[tw] OR "trans people"[tw] OR "trans person*"[tw] OR “gender divers*”[tw] OR “gender affirm*”[tw] OR “gender incongruenc*”[tw] OR “trans people”[tw] OR “trans person”[tw] OR "gender reassignment*"[tw] OR "gender change*"[tw] OR "gender transition*"[tw] OR "trans male*"[tw] “trans masc*”[tw] OR transmasc*[tw] OR "trans female*"[tw] OR transfemin*[tw] OR “trans wom*”[tw] OR “trans man”[tw] OR "gender non-conform*"[tw] OR “gender nonconform*”[tw] OR “gender quest*”[tw] OR "two spirit*"[tw] OR “two-spirit*”[tw] OR "gender fluid*"[tw] OR genderfluid[tw] OR "non-binary"[tw] OR “nonbinary” [tw] OR agender*[tw] OR bigender*[tw] OR intergender[tw] OR “inter-gender”[tw] OR "gender varian*"[tw] OR “gender minorit*”[tw] OR “male to female”[tw] OR “female to male”[tw] OR intersex[tw] OR “inter-sex”[tw] OR multigender[tw] OR “multi-gender”[tw] OR neutrois[tw] OR novidgend*[tw] OR pangend*[tw] OR polygend*[tw] OR “third gender*”[tw] OR "gender queer"[tw] OR genderqueer[tw] OR “gender-queer”[tw])
("Sex Reassignment Procedures"[Mesh] OR "Health Services for Transgender Persons"[Mesh]) OR (“Health service*”[tw] OR “gender affirm*”[tw] OR “gender confirm*”[tw] OR “sex* reassign*”[tw] OR “sex transform*”[tw] OR “gender reassign*”[tw] OR “gender re-assign*”[tw] OR “sex confirm*”[tw] OR “sex affirm*”[tw] OR “recon* surg*”[tw] OR “breast endoprosthesis”[tw] OR “thorax surgery”[tw] OR “prophylactic mastectomy”[tw] OR “simple mastectomy”[tw] OR “partial mastectomy”[tw] OR “subcutaneous mastectomy”[tw] OR “extended radical mastectomy”[tw] OR “modified radical mastectomy”[tw] OR mastectomy[tw] OR mastectomies[tw] OR “chest contour*”[tw] OR “chest-wall contour*”[tw] OR “male genital system surgery”[tw] OR “penile recon*”[tw] OR “penile construct*”[tw] OR “vagina* reconstruction”[tw] OR “vagina* construction”[tw] OR “estrogen therap*”[tw] OR “estrogen replace*”[tw] OR “hormon* therapy”[tw] OR “urolog* surg*”[tw] OR “obstet* surg*”[tw] OR “gyne* surg*”[tw] OR feminization[tw] OR “fac* femin*”[tw] OR masculinization[tw] OR “fac* masculin*”[tw] OR “voice mod*”[tw] OR “tracheal cartilage shave” [tw] OR “otorhinolaryngolog* surg*”[tw] OR “otolaryngolog* surg*”[tw] OR “(Appearance[tw] AND physical[tw] OR facial[tw] OR face[tw] OR brow*[tw] OR cheek*[tw] OR nose[tw] OR nasal[tw] OR lip[tw] OR lips[tw] OR jaw[tw] OR jawline[tw] OR head[tw] OR forehead[tw] OR “frontal sinus”[tw] OR orbit*[tw] OR mandib*[tw] OR “mandibular angle”[tw] OR “upper lip*”[tw] OR hairline[tw] OR thyroid[tw] OR “thyroid cartilage”[tw] OR hair[tw] OR chest[tw] OR breast*[tw] OR nipple*[tw] OR hand*[tw] OR abdomen[tw] OR hips[tw] OR ribs[tw] OR rib[tw] OR ribcage[tw] OR buttocks[tw] OR thigh*[tw] OR leg[tw] OR “upper extremity”[tw] OR “lower extremity”[tw] body*[tw] OR genital*[tw] OR penis[tw] OR penile[tw] OR phallus[tw] OR “glans penis”[tw] scrotum*[tw] OR vagina[tw] OR clitori*[tw] OR vulva[tw] OR labia*[tw] OR perineum[tw] OR perineal[tw] OR body[tw] OR outward[tw] OR gender[tw] OR “gender related” [tw]) OR ("Body image"[Mesh] OR "body image"[tw] OR "body schema*"[tw] OR "body identit*"[tw] OR “body representation”[tw]) OR (“Body dissatisfaction”[Mesh] OR “body dissatisfaction”[tw] OR “appearance dissatisfaction”[tw] OR “body dismorph*”[tw]) OR (“Psychosocial Functioning”[Mesh] OR “Social participation”[Mesh] OR “Emotions”[Mesh] OR “Sexual Dysfunctions, Psychological”[Mesh] OR “Sexual health”[Mesh] OR “Mental health”[Mesh]) OR (“Sex Reassignment Surgery”[Mesh] OR (sex[tw] AND change[tw]) OR (gender[tw] AND (change[tw] OR confirm*[tw] OR affirm*[tw] OR reassignment*[tw])) OR (“Hormone Replacement Therapy”[Mesh] OR “Gonadal Steroid Hormones”[Mesh] OR “Hormones, Hormone Substitutes, and Hormone Antagonists”[Mesh] OR (steroid[tw] AND (sex[tw] OR gender[tw] OR hormone*[tw] OR gonad*[tw]) OR estrogen[tw] OR estradiol[tw] OR progesterone[tw] OR testosterone[tw] OR (hormone*[tw] AND (substitute*[tw] OR antagonist*[tw] OR inhibitor*[tw] OR modulator*[tw] OR agent*[tw] OR replace*[tw] OR therap*[tw] or management[tw])) OR (“Rehabilitation”[Mesh] OR “Activities of Daily Living”[Mesh] OR “Functional Status”[Mesh] OR “Physical functional performance”[Mesh] or “functional performance*”[tw] OR “physical performance*”[tw] OR “physical function*”[tw] OR “daily living*”[tw] OR “daily activit*”[tw] OR “Physical Therapy Modalities”[Mesh] OR physiotherap*[tw] OR “physical therap*”[tw] OR “Occupational Therapy”[Mesh] OR “occupational therap*”[tw] OR “Speech Therapy”[Mesh] OR “Voice Training”[Mesh] OR “Psychotherapy”[Mesh] OR “Person-Centered Psychotherapy”[Mesh] OR “Counseling”[Mesh] OR “Mental Health Services”[Mesh] OR

“Community Health Services”[Mesh] OR “Psychosocial intervention”[Mesh] OR “quality of life”[Mesh] OR “health related quality of life”[tw]) OR “Plastic Surgery”[tw] OR “cosmetic surg*”[tw] OR “esthetic surg*”[tw] OR “plastic surg*”[tw] OR “body contouring”[Mesh] OR “body lift* surg*”[tw] OR “body contour*”[tw] OR “Mammaplasty”[Mesh] OR breast recon* OR mammaplast*[tw] OR mammoplasty*[tw] OR (“breast*”[tw] AND (implant* [tw] OR endoprosthes* [tw] OR augment* [tw])) OR “tissue expan*”[tw] OR thoracic wall surg* OR chest wall surg* OR Mastectomy [Mesh] OR mammectomy*[tw] OR “radical mastectomy*”[tw] OR “chest contour*”[tw] OR “chest-wall contour*”[tw] OR “chest wall contour*”[tw] OR “chest recon*”[tw] OR “breast reduction*”[tw] OR (chest[tw] AND (masculin*[tw] OR femin*[tw])) OR “top surg*”[tw] OR (genital*[tw] AND (masculin*[tw] OR femin*[tw])) OR metoidoplast*[tw] OR (“penile” [tw] AND (construct* [tw] OR recon*[tw])) OR phalloplast*[tw] OR (penis[tw] AND (construct*[tw] OR reconst*[tw])) OR (testicular [tw] AND (construct*[tw] OR reconst*[tw] OR implant[tw] OR prosthetic[tw])) OR (testicle[tw] AND (construct*[tw] OR reconst*[tw] OR implant[tw] OR prosthetic[tw])) OR (“vagin*”[tw] AND (construct*[tw] OR recon* [tw])) OR vaginoplast*[tw] OR neophalloplast*[tw] OR “neo-phalloplast*”[tw] OR neovagin*[tw] OR neo-vagin*[tw] OR labiaplast*[tw] OR “bottom surg*”[tw] OR (fac*[tw] AND (masculin*[tw] OR femin*[tw])) scrotoplast*[tw] OR “voice surg*”[tw] OR “vocal surg*”[tw] OR thyrochondroplast*[tw] OR “tracheal cartilage”[tw] OR hysterectomy*[tw] OR orchiectom*[tw] OR ovariectom*[tw] OR salpingoophorectom*[tw] OR salpingo-oophorect*[tw] OR vaginectom*[tw] OR electrolysis[tw] OR “otolaryngology”[tw] OR castration[tw] OR gonadectomy[tw] OR genioplasty[tw] OR “hair transplant*”[tw] OR “hair procedur*”[tw] or “laser hair removal”[tw] OR “fat grafting”[tw] OR rhinoplast*[tw]
NOT ((“addresses”[Publication Type] OR “biography”[Publication Type] OR “case reports”[Publication Type] OR “comment”[Publication Type] OR “directory”[Publication Type] OR “editorial”[Publication Type] OR “festschrift”[Publication Type] OR “interview”[Publication Type] OR “lectures”[Publication Type] OR “legal cases”[Publication Type] OR “legislation”[Publication Type] OR “letter”[Publication Type] OR “news”[Publication Type] OR “newspaper article”[Publication Type] OR “patient education handout”[Publication Type] OR “popular works”[Publication Type] OR “congresses”[Publication Type] OR “consensus development conference”[Publication Type] OR “consensus development conference, nih”[Publication Type] OR “practice guideline”[Publication Type]) NOT (“animals”[MeSH Terms])

EMBASE
patient-reported outcome.mp. or exp patient-reported outcome/ or (outcome assessment.mp. or exp outcome assessment/) or (quality of life.mp. or exp "quality of life"/) or (hr-pro or hrpro or hrql or HRQOL or ql or qol or life quality).mp. or (health index or health indices or health profile* or health status*).mp. or (appraisal* or appraised or report or reported or reporting or rated or rating* or based or assessed or assessment*).mp. or (disability or function or functional or functions or subjective or utility or utilities or wellbeing or 'well being').mp. or (index or indices or instrument or instruments or measure or measures or questionnaire* or profile or profiles or scale or scales or score or scores or status or survey or surveys).mp. OR exp health survey/ OR exp health care survey/ OR exp short survey/ OR exp health services research/ OR exp implementation science/ or implementation.mp.
transgender*.tw. OR transsexual*.tw. OR ("trans m#n" or "trans wom#n" or "trans person*" or transpeople or "trans male*" or "trans female*").tw. OR ((gender adj3 (non-binary or nonbinary or bigender or agender or fluid* or trans* or non-conform* or nonconform* or genderqueer)) or divers* or incongruenc* or affirm*) or gender-nonconform*.tw. OR gender dysphori*.mp. OR two-spirit.mp. OR two spirit.mp. OR trigend*.mp. OR tri-gend*.mp. OR third gender.mp. OR gender euphoria.tw. OR (sex reassignment* or gender reassignment* or gender affirm*).tw. OR (transfeminine or transmasculine).tw. OR (AFAB or AMAB).tw. OR (gender adj (expression* or transition*)).tw. OR (transvestite* or transvestism).tw. OR exp transgender/ OR exp transsexualism/ OR exp sex reassignment/ OR exp sex transformation/ OR exp male to female transgender/ OR exp female to male transgender/ OR exp gender dysphoria/ OR ((intergender or inter gender or intersex or inter-sex or multigender or multi-gender or neutrois or novigend* or pangend* or polygend*).tw.)
exp physical appearance/ or exp body image/ or body image.mp. or (appearance adj3 (head or hair or hairline or frontal or facial or face or eyebrow or eye or orbit or nose or forehead or mandib* or nasal or lips or lip or cheek* or jawline or jaw or shoulders or chest or breast* or nipple* or hand* or feet or abdomen or hips or ribs or ribs or ribcage or buttocks or thigh* or leg or upper extremity or lower extremity or body* or genital* or penis or penile or phallus or glans penis or glans or scrot* or vagina or vulva or labia* or perineum or perineal or outward or gender or gender related)).mp. or health-related quality of life.mp. or exp "quality of life"/ or exp wellbeing/ or exp psychological well-being/ or exp mental health/ or exp wellbeing/ or psychosocial.mp. or exp psychosocial rehabilitation/ or exp psychosocial care/ or sexual function.mp. or exp sexual function/ or exp patient counseling/ or counseling.mp. or exp sexual counseling/ or rehabilitation.mp. or exp community based rehabilitation/ or exp rehabilitation/ or exp rehabilitation patient/ or exp speech rehabilitation/ or exp "speech and language rehabilitation"/ or exp rehabilitation care/ or exp psychosocial rehabilitation/ or physiotherapy.mp. or exp physiotherapy/ or occupational therapy.mp. or exp occupational therapy/ or voice.mp. or exp voice analysis/ or exp voice/ or voice prosthesis/ or voice training/ or exp voice change/ or hormone replacement.mp. or exp hormone substitution/ or steroid hormone/ or exp sex hormone/ or exp hormone inhibition/ or exp "silencing mediator of retinoid and thyroid hormone receptor"/ or exp hormone antagonist/ or reconstructive surgery.mp. or exp reconstructive surgery/ or exp plastic surgery/ or cosmetic surgery.mp. or exp esthetic surgery/ or (femin* adj3 (head or hair or hairline or facial or face or forehead or frontal or eyebrow or eye or orbit or nose or nasal or lips or lip or jawline or jaw or mandible or mandibular or shoulders or chest or breast* or nipple* or hand* or abdomen or hips or ribs or ribs or ribcage or buttocks or thigh* or leg or upper extremity or lower extremity or body* or genital* or outward or gender)).mp. or (masculin* adj3 (head or hair or hairline or facial or face or forehead or frontal or eyebrow or eye or orbit or nose or nasal or lips or lip or jawline or jaw or mandible or mandibular or shoulders or chest or breast* or nipple* or hand* or abdomen or hips or ribs or ribs or ribcage or buttocks or thigh* or leg or upper extremity or lower extremity or body* or genital* or outward or gender)).mp. or (viriliz* adj3 (head or hair or hairline or facial or face or forehead or frontal or eyebrow or eye or orbit or nose or nasal or lips or lip or jawline or jaw or mandible or mandibular or shoulders or chest or breast* or nipple* or hand* or abdomen or hips or ribs or ribs or ribcage or buttocks or thigh* or leg or upper extremity or lower extremity or body* or genital* or outward or gender)).mp. or (reconst* adj3 (head or hair or hairline or facial or face or forehead or frontal or eyebrow or eye or orbit or nose or nasal or lips or lip or jawline or jaw or mandible or mandibular or shoulders or chest or breast* or nipple* or hand* or abdomen or hips or ribs or ribs or ribcage or buttocks or thigh* or leg or upper extremity or lower extremity or body* or genital* or outward or gender)).mp. or (construct* adj3 (head or hair or hairline or facial or face or forehead or frontal or eyebrow or eye or orbit or nose or nasal or lips or lip or jawline or jaw or mandible or mandibular or shoulders or chest or breast* or nipple* or hand* or abdomen or hips or ribs or ribs or ribcage or buttocks or thigh* or leg or upper extremity or lower extremity or body* or genital* or penile or vaginal or outward or gender)).mp. or (breast* adj (augment* or implant* or reduction* or lift* or endoprosthes*)).mp. or (mastectom* or mammaplast* or mammoplast*).mp. or (top surg* or bottom surg* or phalloplast* or scrotoplast* or glans plast* or glansplast* or erectile device* or vaginoplast* or neophalloplast* or neo-phalloplast* or neovagin* or labiaplast* or urolog* surg* or obstet* surg* or gyne* surg* or voice surg* or vocal surg* or thyrochondroplast* or tracheal cartilage shave or hysterectomy* or orchiectom* or ovariectom* or salpingoophorectom* or vaginectom* or electrolysis or otorhinolaryngolog* surg* or otolaryngolog* surg* or castration or gonadectomy or laryngoplast* or

rhinoplast* or geniolast* or hair* advanc* or hair* transplant* or laser hair removal or testicul* implant* or testicul* prosthetic*).mp.

MEDLINE
exp Surveys and Questionnaires/ or exp patient reported outcome measures/ or patient reported outcome measur*.mp. or patient-reported outcome.mp. or outcome assessment.mp. or HR-PRO.mp. or HRPRO.mp. or HRQL.mp. or HRQOL.mp. or QL.mp. or QOL.mp. or quality of life.mp. or function*.mp. or satisfaction.mp. or index.mp. or indices.mp. or instrument*.mp. or measure*.mp. or PROM.mp. or survey*.mp. or questionnaire*.mp. or outcome assessment.mp. or exp Health Surveys/ or exp Health Care Surveys/ or exp Outcome Assessment, Health Care/ OR exp health services administration/ OR exp implementation science/ OR patient education.mp OR implementation.mp OR exp delivery of health care/ OR exp evaluation research/ OR decision making.mp. OR framework.mp. OR diffusion of innovation.mp.
exp Sexual and Gender Minorities/ OR (exp Transgender Persons/ or exp Transsexualism/ or exp Gender Dysphoria/ or exp Disorders of Sex Development/ or exp Gender Identity/ or transgender*.mp. or transsexual*.mp. or transvestite*.mp. or transvestism.mp. or gender dysphoria.mp. or gender euphoria.mp. or MTF.mp. or FTM.mp. or trans m#n.mp. or trans wom#n.mp. or trans person*.mp. or transpeople.mp. or trans male*.mp. or trans female*.mp. or nonbinary.mp. or non-binary.mp. or genderqueer.mp. or gender-queer.mp. or agender.mp. or bigender.mp. or gender fluid.mp. or gender var*.mp. or two-spirit.mp. or two spirit.mp. or gender nonconform*.mp. or gender quest*.mp. or gender varian*.mp. or genderfluid.mp. or intergender.mp. or inter gender.mp. or intersex.mp. or inter sex.mp. or inter-sex.mp. or multigender.mp. or multi-gender.mp. or neutrois.mp. or novigend*.mp. or pangend*.mp. or polygend*.mp. or third gender.mp. or transfemin*.mp. or third gend*.mp. or transmasc*.mp. or trans fem*.mp. or trans masc*.mp. or transmasc*.mp. or transfemin* trigend*.mp. or AFAB.mp. or AMAB.mp. or gender expression.mp. or gender transition.mp. or gender identity.mp. or tri gend*.mp.) OR (gender adj3 (non-binary or agender or fluid* or trans* or non-conform* or nonconform*)).mp
exp Health Services/ or exp Health Services for Transgender Persons/ or health services for transgender persons.mp. or exp Sex Reassignment Procedures/ or exp Sex Reassignment Surgery/ or health service*.mp. or gender affirm*.mp. or gender confirm*.mp. or sex reassign*.mp. or sex transform*.mp. or gender reassign.mp. or gender re-assign*.mp. or sex confirm*.mp. or sex affirm*.mp. or exp Reconstructive Surgical Procedures/ or reconstructive surg*.mp. or exp Surgery, Plastic/ or plastic surg*.mp. or exp body contouring/ or body contour*.mp. or breast recon*.mp. or exp Mammaplasty/ or exp Breast Implants/ or exp Breast Implantation/ or mammaplasty.mp. or mammoplasty.mp. or breast implant*.mp. or breast augment*.mp. or tissue expan*.mp. or thoracic wall surg*.mp. or chest wall surg*.mp. or mastectomy.mp. or chest contour*.mp. or chest-wall contour*.mp. or chest wall contour*.mp. or reduction mammoplasty.mp. or reduction mammaplasty.mp. or mastoplasty.mp. or mastectomy.mp. or mastectomies.mp. or exp Mammaplasty/ or exp Mastectomy, Simple/ or exp Mastectomy, Segmental/ or exp Mastectomy, Modified Radical/ or exp Mastectomy/ or exp Mastectomy, Extended Radical/ or exp Mastectomy, Subcutaneous/ or exp Mastectomy, Radical/ or metoidoplast* or penile recon* or penile construct* or phalloplast* or vagina* reconstruct* or vaginoplast* or vagina* construct* or neophalloplast* or neo-phalloplast* or neovagina* or neo-vagin* or genital reassign* or genital re-assign* or exp Otolaryngology/ or otorhinolaryngolog* surg*.mp. or otolaryngolog* surg*.mp. or exp Estrogen Replacement Therapy/ or exp Hormones/ or exp Steroids/ or exp Urologic Surgical Procedures/ or estrogen replace*.mp. or testosterone therap*.mp. or urolog* surg*.mp. or obstet* surg*.mp. or gyne* surg*.mp. or fac* femin*.mp. or masculinization.mp. or top surg*.mp. or bottom surg*.mp. or fac* masculin*.mp. or exp Feminization/ or scrotoplast*.mp. or voice surg*.mp. or thyrochondroplast*.mp. or tracheal cartilage shave.mp. or exp Hysterectomy, Vaginal/ or exp Hysterectomy/ or exp orchiectomy/ or exp ovariectomy/ or exp salpingo-oophorectomy/ or vaginectomy.mp. or exp obstetric surgical procedures/ or exp Gynecologic Surgical Procedures/ or exp electrolysis/ or hysterectomy.mp. or orchiectom*.mp. or ovariectom*.mp. or salpingo-oophorect*.mp. or salpingoophorectom*.mp. or vaginectom*.mp. or voice mod*.mp. or exp electrolysis/ or electrolysis.mp.

PSYCINFO
exp Surveys/ OR exp Questionnaires/ OR (exp "Quality of Life"/ or exp Treatment Outcomes/ or exp Self-Report/ or exp Psychometrics/ or exp Patient Reported Outcome Measures/ or Measurement/) OR (hr-pro or hrpro or hrql or hrqol or ql or qol or life quality or satisfaction or health index or health indices or health profile* or health status).mp. OR (apprais* or report or reported or reporting or rated or rating* or based or assessed or assessment*).mp. OR (disability or function or functional or functions or subjective or utility or utilities or wellbeing or well being).mp. OR (index or indices or instrument or instruments or measure or measures or questionnaire* or profile or profiles or scale or scales or score or scores or status or survey or surveys or self-report or patient reported outcome measure* or patient-reported outcome measure or PROM*).mp. OR implementation.mp.
exp Health Care Delivery/ or exp Health Care Utilization/ or exp Health Care Services/ or exp Gender Reassignment/ or exp Surgery/ or exp Plastic Surgery/ OR exp Cosmetic Techniques/ or exp Mastectomy/ OR exp Drug Therapy/ or exp Steroids/ or exp Prostheses/ or exp Hysterectomy/ or exp Ovariectomy/ OR (health service* or gender affirm* or gender confirm* or sex reassign* or sex transform* or gender reassign or gender re-assign* or sex confirm* or sex affirm*) OR (reconstructive surg* or plastic surg*).mp. or exp body contouring/ or body contour*.mp. or breast recon*.mp. or mammaplasty.mp. or mammoplasty.mp. or breast implant*.mp. or breast augment*.mp. or tissue expan*.mp. or thoracic wall surg*.mp. or chest wall surg*.mp. or mastectomy.mp. or chest contour*.mp. or chest-wall contour*.mp. or chest wall contour*.mp. or reduction mammoplasty.mp. or reduction mammaplasty.mp. or mastoplasty.mp. or mastectomy.mp. or mastectomies.mp. or metoidoplast*.mp. or penile recon*.mp. or penile construct*.mp. or phalloplast*.mp. or vagina* reconstruct*.mp. or vaginoplast*.mp. or vagina* construct*.mp. or neophalloplast*.mp. or neo-phalloplast*.mp. or neovagina*.mp. or neo-vagin*.mp. or genital reassign*.mp. or genital re-assign*.mp. OR (otorhinolaryngolog* surg* or otolaryngolog* surg* or estrogen replace* or testosterone therap* or urolog* surg* or obstet* surg* or gyne* surg* or fac* femin* or masculinization or top surg* or bottom surg* or fac* masculin* or scrotoplast* or voice surg* or thyrochondroplast* or tracheal cartilage shave or vaginectomy or hysterectomy or orchiectom* or ovariectom* or salpingo-oophorect* or salpingoophorectom* or vaginectom* or voice mod* or electrolysis).mp.
exp Transgender/ or exp Gender Identity/ or exp Transsexualism/ or exp Gender Dysphoria/ or "Transgender (Attitudes Toward)"/ or exp Gender Reassignment/ or (trans sexual or transexual or trans m#n or trans wom#n or trans person*).mp. OR (transpeople or trans male* or trans female or sex change* or Gender Nonconform* or gender non-conform* or Gender identity or transsexualism or gender identity or non-binary or agender or bigender or trigender or tri gender or nonbinary or genderfluid* or genderqueer or gender queer or gender varian* or gender ambigui* or cross gender* or two-spirit or two spirit or gender quest* or intergender or inter gender or inter-gender or intersex or inter-sex or inter sex or multi gender or multi-gender or neutrois or novigend* or polygend* or third gender or trans masc* or transmasc* or transfemin* or trans fem*).mp. OR ((Gender adj3 (non-binary or agender or fluid* or non-conform* or nonconform*)).mp. OR exp Gender Nonconforming/ or gender euphor*.mp.)

CINAHL
(MH "Patient-Reported Outcomes") OR (MH "Outcome Assessment") OR (MH "Quality of Life+") OR (MH questionnaire or survey or scale or instrument or measurement or measure) OR ("TI(“hr-pro” OR “hrpro” OR “hrql” OR “hrqol” OR “ql” OR “qol” OR “quality of life” OR “life quality” OR “health index*” OR “health indices” OR “health profile*” OR “health status”) OR AB(“hr-pro” OR “hrpro” OR “hrql” OR “hrqol” OR “ql” OR “qol” OR “quality of life” OR “life quality” OR “health index*” OR “health indices” OR “health profile*” OR “health status”)OR TI(((patient OR self OR child OR parent OR carer OR proxy) N2 (appraisal* OR appraised OR report OR reported OR reporting OR rated OR rating* OR based OR assessed OR assessment*))) OR AB (((patient OR self OR child OR parent OR carer OR proxy) N2 (appraisal* OR appraised OR report OR reported OR reporting OR rated OR rating* OR based OR assessed OR assessment*))) OR TI (((disability OR function OR functional OR functions OR subjective OR utility OR utilities OR wellbeing OR “well being”) N2 (index OR indices OR instrument OR instruments OR measure OR measures OR questionnaire* OR profile OR profiles OR scale OR scales OR score OR scores OR status OR survey OR surveys))) OR AB (((disability OR function OR functional OR functions OR subjective OR utility OR utilities OR wellbeing OR “well being”) N2 (index OR indices OR instrument OR instruments OR measure OR measures OR questionnaire* OR profile OR profiles OR scale OR scales OR score OR scores OR status OR survey OR surveys OR PROM* OR implementation OR “patient-reported outcome measure*” OR “patient reported outcome measure*”))))
(MH mental health treatment or mental health services or mental health care) OR (MH psychotherapy or therapy or counseling or intervention or treatment) OR (TX psychosocial interventions or strategies or best practices or treatment or therapy) OR (MH speech therapy or treatment or intervention) OR (MH voice therapy or voice treatment or voice training or voice modification) OR TX appearance N3 (head or hair or hairline or facial or face or forehead or frontal or eyebrow or eye or orbit or nose or nasal or lips or lip or jawline or jaw or mandib* or cheek* or shoulder* or chest or breast or nipple or hands or upper extremity or abdomen or hips or or ribs or ribcage or buttocks or thigh or leg or back or lower extremity or body or feet or genital* or penis or penile or phallus or glans penis or glans or scrot* or vagin* or vulva or labia or perineum or perineal or outward or gender or gender related or gender-related) OR TX reconstr* N3 (head or hair or hairline or facial or face or forehead or frontal or eyebrow or eye or orbit or nose or nasal or lips or lip or jawline or jaw or mandib* or cheek* or shoulder* or chest or breast or nipple or hands or upper extremity or abdomen or hips or or ribs or ribcage or buttocks or thigh or leg or back or lower extremity or body or feet or genital* or penis or penile or phallus or glans penis or glans or scrot* or vagin* or vulva or labia or perineum or perineal or outward or gender or gender related or gender-related) OR TX construct* (head or hair or hairline or facial or face or forehead or frontal or eyebrow or eye or orbit or nose or nasal or lips or lip or jawline or jaw or mandib* or cheek* or shoulder* or chest or breast or nipple or hands or upper extremity or abdomen or hips or or ribs or ribcage or buttocks or thigh or leg or back or lower extremity or body or feet or genital* or penis or penile or phallus or glans penis or glans or scrot* or vagin* or vulva or labia or perineum or perineal or outward or gender or gender related or gender-related) OR TX hormone blockers OR TX (hormone replacement therapy or hrt or hormone therapy) OR TX puberty blockers OR TX puberty hormones OR TX estrogen replacement therapy OR TX (testosterone replacement therapy or trt) OR TX estrogen replacement therapy OR TX (sex hormones or estrogen or progesterone or estradiol) OR TX hormone substitution OR TX steroid hormones OR TX hormone antagonist OR TX antiandrogen drugs OR TX spironolactone OR TX plastic surgery or cosmetic surgery or cosmetics procedures OR esthetic surgery or body contouring or mammaplasty or breast reconstruction or mammoplasty or breast implant or breast prosthesis or breast augmentation or radical mastectomy or chest contouring or chest contour or breast reduction or chest wall contour or chest reconstruction or chest masculinization or chest feminization or top surgery or bottom surgery or genital masculinization or gential feminization or genital gender affirming surgery or genital gender affirming procedures or metoidioplasty or phalloplasty or male genital reconstruction or penile reconstruction or penis reconstruction or testicular reconstruction or testicular construction or testicle construction or erectile device or erectile implant or penile prosthesis or penile implant or scrotoplasty or glansplasty or neophalloplasty or vaginal reconstruction or vaginal construction or vaginectomy or labiaplasty or clitoral reduction or clitoris or neovaginoplasty or urology or urological surgery or obstetric surgery or gynecologic surgery or facial masculinization or facial feminization or voice surgery or vocal surgery or vocal chords surgery or thyrochondroplasty or tracheal cartilage shave or hysterectomy or orchiectomy or ovariectomy or saphingoopherectomy or otorhinolaryngology or castration or gonadectomy or gemiolasty or hair transplant or hair advancement or laser hair removal or fat grafting or rhinoplasty
(MH "Transgender Persons+") OR (MH "Transsexuals") OR (MH "Transsexualism") OR (MH "Sex Reassignment Procedures+") OR (MH “Gender Identity”) OR (MH “Gender Dysphoria”) OR TX (trans sexual or transexual or sex change) or OR TX transgender* OR TX transsexual* OR TX "trans m#n" or "trans wom#n" or "trans person*" or transpeople or "trans male*" or "trans female*" OR TX gender* N3 (non-binary or fluid* or trans* or non-conform* or nonconform* or queer or gender varian* or gender ambigui* or cross gender* or two-spirit or two spirit or gender quest* or intergender or inter gender or inter-gender or intersex or inter-sex or inter sex or multi gender or multi-

gender or neutrois or novigend* or polygend* or third gender or trans masc* or transmasc* or gender identity or transsexualism or non-binary or agender or bigender or trigender or tri gender or genderfluid* or gender queer or transfemin* or trans fem* or diverse* or incongru* or affirm*) OR TX gender dysphori* OR TX sex reassignment* or gender reassignment* or gender affirm* OR TX transfeminine or transmasculine OR TX AFAB or AMAB OR TX gender N2 (expression* or transition*) OR TX gender minorit* or gender diver*

WEB OF SCIENCE
TS=(“questionnaire*” or “survey*” or “scale” or “instrument*” or “measurement” or “measure*” or “quality of life” or “life quality” or “health index*” or “health indices” or “health profile*” or “health status" or “appraisal*” or “appraised” or “report*” or “rated” or “rating*” or “based” or “assessed” or “assessment*” or “function” or “functional” or “functions” or “satisfaction” or “implantation” or “health services” or “subjective” or “utility” or “utilities” or “wellbeing” or “well being” or “index” or “indices” or “instrument” or “instruments” or “measure*” or “questionnaire*” or “profile” or “profiles” or “scale*” or “score*” or “status” or “survey*” or “PROM*” or “patient-reported outcome measure*” or “patient reported outcome measure*”)
TS=(“health service*” or “gender affirmation procedures” or “gender affirm*” or “gender confirm*” or “sex reassign*” or “sex transform*” or “gender reassign*” or “gender re-assign” or “sex confirm*” or “sex affirm*” or “gender affirmation surgery” or “gender affirming” or “surgery” or “plastic surg*” or “reconstructive surg*” or “craniofacial recon*” or “breast recon*” or “mammaplast*” or “mammoplast*” or “breast implant” or “breast augment*” or “tissue expan*” or “thoracic wall surg*” or “mastectom*” or “reduction mammoplast*” or “reduction mammaplast*” or “mastoplasty” or “metoidoplast*” or “penile recon*” or “penile construct*” or “phalloplast*” or “vagina* reconstruct*” or “vagina* construct*” or “neophalloplast*” or “neo-phalloplast*” or “neovagina” or “neo-vagina” or “genital reassign” or “genital re-assign”“otorhinolaryngologic surg*” or “otolaryngologic surg*” or “head and neck surgery” or “body contour*” or “prosthesis” or “implant*” or “breast implant*” or “penile prosthesis” or “mastectom*” or “gyne* surg*” or “obstet* surg*” or “urolog* surg*” or “hormone* therap*” or “estrogen” or “testosterone” or “steroids” or “chest wall surg*” or “chest-wall surg*” or “voice therapy” or “feminization” or “hysterectomy” or “oophorectomy” or “orchiectomy” or “electrolysis” or “face feminin*” or “masculinization” or “top surg*” or “bottom surg*” or “fac* masculin*” or “scrotoplast*” or “voice surg*” or “thyrochondroplast*” or “tracheal cartilage shave” or “vaginectomy” or “hysterectomy” or “orchiectomy*” or “ovariectomy*” or “salpingo-oophorect*” or “salpingoophorectom*” or “vaginectom*” or “voice mod” or “electrolysis”)
TS=(“Transgender*” or “transsexual*” or “sex reassign*” or “gender identity” or “gender dysphoria” or “trans sexual” or “trans m#n” or “trans wom#n” or “trans person” or “transpeople” or “trans male” or “trans female” or “sex change*” or “gender nonconform*” or “gender non-conform*” or “gener nonbinary” or “non-binary” or “nonbinary” or “agender” or “bigender” or “trigender” or “tri gender” or “gender fluid” or “genderfluid” or “genderqueer” or “gender queer” or “intergender” or “inter gender” or “inter-gender” or “intersex” or “inter-sex” or “inter sex” or “multi gender” or “multi-gender” or “neutrois” or “novigend*” or “polygend*” or “third gender*” or “trans masc*” or “transmasc*” or “transfemin*” or “trans fem*” or “AFAB” or “AMAB” or “transvestit*” or “gender minorit*” or “gender diver*”)

Grey Literature Search Strategy	
**Database, Search Engine, or Website URL**	**Search Strategy**
Opengrey.eu	(Patient-reported outcome measure OR Questionnaire) AND (Transgender OR Nonbinary) AND (Gender-affirming care)
New York Academy of Medicine's Grey Literature Report (http://www.greylit.org/home)	(Patient-reported outcome measure OR Questionnaire) AND (Transgender OR Nonbinary) AND (Gender-affirming care)
National Institute for Health and Care Excellence Guidance (https://www.nice.org.uk/guidance)	(Patient-reported outcome measure OR Questionnaire) AND (Transgender OR Nonbinary) AND (Gender-affirming care)
Google.com (first 10 pages)	(Patient-reported outcome measure OR Questionnaire) AND (Transgender OR Nonbinary) AND (Gender-affirming care)
Google.co.uk (first 10 pages)	(Patient-reported outcome measure OR Questionnaire) AND (Transgender OR Nonbinary) AND (Gender-affirming care)
Gender Identity Research and Education Society (https://www.gires.org.uk/)	(Patient-reported outcome measure OR Questionnaire) AND (Transgender OR Nonbinary) AND (Gender-affirming care)
Gendered Intelligence (https://genderedintelligence.co.uk/)	(Patient-reported outcome measure OR Questionnaire) AND (Transgender OR Nonbinary) AND (Gender-affirming care)
Stonewall UK (https://www.stonewall.org.uk/)	(Patient-reported outcome measure OR Questionnaire) AND (Transgender OR Nonbinary) AND (Gender-affirming care)

Appendix 2. Age for patients included in the review categorised by study

Study	Mean Age of Patients Included	SD	Minimum Age	Maximum Age
McGuire et al., 2021[27]	14.9	1.85	11.1	17
De Vries et al., 2014[28]	13.6	NR	NR	NR
Meyenburg et al., 2015[29]	21.3	NR	11	35
Nieder et al., 2021[30]	17.4	1.7	11	21
Becker-Hebly et al., 2021[31]	15.6	1.2	NR	NR
Pauli et al., 2020[33]	16.3	NR	13.7	20.2
Arnoldussen et al., 2020[35]	14.6	NR	10.1	18.1
De Vries et al., 2011[36]	13.65	1.85	NR	NR
Costa et al., 2015[37]	15.52	NR	12	17
Peterson et al., 2020[38]	17.04	NR	11	24
Achille et al., 2020[39]	16.2	2.2	NR	NR
Grannis et al., 2021[40]	16.39	NR	NR	NR
De Vries et al., 2016[41]	15.805	NR	NR	NR
Quinn et al., 2018[42]	17	NR	NR	NR
De Graaf et al., 2018[43]	14.875	NR	NR	NR
Kuper et al., 2020[44]	14.9	NR	9	18
O’Bryan et al., 2020[45]	17.1	NR	8	21
Bowen et al., 2021[46]	15.4	NR	13	16
Kuper et al., 2019[47]	15.3	1.52	12	18
Fontanari et al., 2020[48]	18.61	NR	16	24

Appendix 3. CASP Checklist Results for Included Articles

CASP Checklist Item	Rating		
	**Yes**	**No**	**Can't Tell**
Cohort Studies Checklist (n=20)			
Did the study address a clearly focused issue?	20	0	0
Was the cohort recruited in an acceptable way?	20	0	0
Was the exposure accurately measured to minimise bias?	0	20	0
Was the outcome accurately measured to minimise bias?	20	0	0
Have the authors identified all important confounding factors?	20	0	0
Have they taken account of the confounding factors in the design and/or analysis?	0	20	0
Was the follow up of subjects complete enough?	10	10	0
Was the follow up of subjects long enough?	10	10	0
Do you believe the results?	20	0	0
Can the results be applied to the local population?	20	0	0
Do the results of this study fit with other available evidence?	20	0	0
